# Functional test of PCDHB11, the most human-specific neuronal surface protein

**DOI:** 10.1186/s12862-016-0652-x

**Published:** 2016-04-12

**Authors:** Guilherme Braga de Freitas, Rafaella Araújo Gonçalves, Matthias Gralle

**Affiliations:** Instituto de Bioquímica Médica Leopoldo de Meis, Universidade Federal do Rio de Janeiro, Rio de Janeiro, Brazil

**Keywords:** Human evolution, Neutral evolution, Gene ontology, Clustered protocadherins

## Abstract

**Background:**

Brain-expressed proteins that have undergone functional change during human evolution may contribute to human cognitive capacities, and may also leave us vulnerable to specifically human diseases, such as schizophrenia, autism or Alzheimer’s disease. In order to search systematically for those proteins that have changed the most during human evolution and that might contribute to brain function and pathology, all proteins with orthologs in chimpanzee, orangutan and rhesus macaque and annotated as being expressed on the surface of cells in the human central nervous system were ordered by the number of human-specific amino acid differences that are fixed in modern populations.

**Results:**

PCDHB11, a beta-protocadherin homologous to murine cell adhesion proteins, stood out with 12 substitutions and maintained its lead after normalizing for protein size and applying weights for amino acid exchange probabilities. Human PCDHB11 was found to cause homophilic cell adhesion, but at lower levels than shown for other clustered protocadherins. Homophilic adhesion caused by a PCDHB11 with reversion of human-specific changes was as low as for modern human PCDHB11; while neither human nor reverted PCDHB11 adhered to controls, they did adhere to each other. A loss of function in PCDHB11 is unlikely because intra-human variability did not increase relative to the other human beta-protocadherins.

**Conclusions:**

The brain-expressed protein with the highest number of human-specific substitutions is *PCDHB11.* In spite of its fast evolution and low intra-human variability, cell-based tests on the only proposed function for PCDHB11 did not indicate a functional change.

## Background

Human brains are different from other primate brains. However, it is not clear if the difference is simply a matter of size [1] or if there are any molecular and cellular differences that would help explain uniquely human capabilities. One molecular difference that might be important for the acquisition of such a capability, namely of speech, is the substitution of two amino acids in the protein FOXP2 [2–4]. Other human-specific differences have been discovered in proteins expressed in the brain, but have not yet been linked to a behavioral phenotype [5–8]⁠.

It has been surmised for more than 30 years that the main genetic differences between humans and chimpanzees lie in regulatory sequences [9], and recent studies have begun to identify some of the more relevant human-specific elements [10–13]. However, even with the increasing number of sequenced human and other primate genomes, it remains more difficult to identify functionally relevant differences in non-coding parts of the genome, even in well-studied transcription factor binding sites [14], than in protein-coding sequences. Therefore, the present study concentrates on differences in the protein-coding parts of the genome.

While the genomes of individuals from extinct human populations are exciting sources of information on human evolution [15–19], we lack reliable information on the cognitive capabilities of those populations [20, 21]. Therefore, the most useful comparison is with living primates, on whose cognitive phenotype we do have information [22, 23]: a DNA variant might contribute to human cognitive capabilities if it is present in all cognitively normal modern humans, but not in the aligned genomes of other primates. The analysis of primate genomes has yielded lists of such variants [24–27]; however, the statistical tests for positive selection are necessarily of low statistical power and selectivity, and the necessary biochemical analysis of such candidate genes has rarely been reported [28].

The initial auto-organization of the human brain and its subsequent forming by the environment are mediated by cell surface proteins [29–31]. Variation in adhesion proteins has been shown to degrade [32] and, possibly, to explain part of normal variation in cognitive function [33]. While the example of FOXP2 shows that intracellular proteins, such as transcription factors, may be important targets of change, the function of proteins present at the cell membrane, such as receptors, channels and adhesion proteins, is more straightforward to quantify. Therefore, the bioinformatical strategy used in the present study was to rank all proteins present on the surface of human central nervous system cells by the number of substitutions they have accumulated on the human lineage. The aim of this strategy was to select a neural cell surface protein with high probability of having changed its function on the human lineage, so that this functional change could be tested for in biological model systems.

## Results

### Selection of candidate protein

**Fig. 1:**
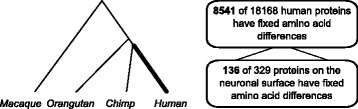
Pipeline for discovering human-specific amino acid substitutions. Amino acids in human proteins were considered human-specific wherever they differed from the consensus between the exomes of *Macaca mulatta, Pongo abelii* and *Pan troglodytes.* Differences were considered fixed if the human-specific amino acid recurred in 100 haploid human genomes. Among those proteins that could be aligned between the four genomes, the indicated number of proteins contains at least one fixed human-specific difference

A comparison of the protein-coding regions of the reference chimpanzee, orangutan and rhesus macaque genomes to 100 haploid human genomes, sampled from diverse human populations, has been published before [34] and resulted in a list of amino acid positions where all modern human genomes agree with each other and are different from the non-human primates. Almost half of all human proteins contain at least one such amino acid (Fig. 1).

The present study focuses on the substitutions occurring in brain cell-surface proteins, i.e. the products of genes annotated both as being expressed in central nervous system cells and as present on the extracellular side of the plasma membrane, according to Gene Ontology [35]. Among 329 proteins in this set, 136 contain at least one fixed human-specific difference (Fig. 1). An unknown fraction of these human-specific substitutions may have had functional consequences. While ideally the functional consequences might be estimated from the position of a substitution within the three-dimensional structure of a protein, especially if structure-function relationships are well established, such structural data are not available for many of the candidate proteins. Alternatively, reasoning that a change in function may require several amino acid substitutions or that a change, once it has occurred, may release a functional restraint and permit additional substitutions to occur, the 136 candidate proteins were ordered by the number of fixed human-specific amino acid differences, with β-protocadherin 11 (PCDHB11) appearing at the top of the list, due to its 12 substitutions (Table 1).

**Table 1:** Proteins on the surface of central nervous system cells that have accumulated the highest number of amino acid substitutions on the human lineage

Protein	Substitutions	Length of protein	Subst./length	Weighted subst.	Weighted subst./length
PCDHB11	12	797	0.015	89	0.112
ICAM1	6	532	0.011	41	0.077
HTR3E	4	456	0.009	33	0.072
HRH1	5	487	0.010	31	0.064
DRD5	5	477	0.010	30	0.063
PCDHB13	6	798	0.008	47	0.059
GLRA4	4	417	0.010	24	0.058
HOME3	2	361	0.006	19	0.053
PCDHB6	5	794	0.006	40	0.050
VIPR1	2	457	0.004	20	0.044
CCKAR	2	428	0.005	18	0.042
PCDHB12	4	797	0.005	30	0.038
OXYR	3	389	0.008	14	0.036
GRIN3A	6	1115	0.005	38	0.034
PCDHB10	3	797	0.004	27	0.034
SEMA5B	5	1151	0.004	32	0.028
PCDHA5	4	936	0.004	26	0.028
PCDHB14	4	798	0.005	21	0.026
GRIN2C	4	1236	0.003	32	0.026
PCDHA2	4	948	0.004	23	0.024
CD44	3	742	0.004	17	0.023
PCDHB15	4	787	0.005	18	0.023
PCDH15	9	1955	0.005	43	0.022

For each protein, the number of human-specific substitutions, divided by the length of the protein, gives the fraction of amino acids that have changed and become fixed on the human lineage. For each amino acid substitution on the human lineage, a weight was derived from the BLOSUM100 matrix that reflects the rareness of this exchange between organisms that have 99 % amino acid identity, and the weights of all substitutions were summed up for each protein

While a high number of substitutions does not necessarily indicate a change in function, several aspects make PCDHB11 stand out from other proteins on the list. Higher numbers of substitutions would be expected to occur by chance in longer proteins; the absolute number was therefore divided by the length of the protein to exclude this explanation. The high rate of substitutions in several proteins, such as PCDH15, can readily be explained by their large size, but PCDHB11 continues to stand out (Table 1). Furthermore, in exome comparisons, some amino acids are frequently found in substitution for each other, probably because their exchange has a lower impact on the function of the protein. The rate of amino acid exchange, when comparing proteins in closely related species, was used in order to weigh the importance of an exchange; amino acids that rarely substitute for each other were given higher weights. When summing up the weights for all the substitutions, PCDHB11 continues to have a score well above those of all the other candidate proteins (Table 1), due to several evolutionarily rare amino acid exchanges, e.g. asparagine to isoleucine and arginine to isoleucine (Table 2).

**Table 2:** Differences between non-human primate consensus and human PCDHB11

Position	Consensus	Human	Weight	Domain
4	Glu	Gln	3	Signal peptide
106	Phe	Leu	5	EC1^a^
134	Leu	Ser	11	EC2^a^
185	Asn	Ile	12	EC2^a^
213	Thr	Ser	3	EC2^a^
252	Pro	Arg	10	EC3^a^
263	Thr	Ile	8	EC3^a^
281	Leu	Phe	5	EC3^a^
304	Thr	Arg	8	EC3^a^
334	Arg	Ile	12	EC3^a^
336	Gln	His	4	EC3^a^
724	Arg	Ser	8	Cytoplasmic

Differences between modern human PCDHB11 and the consensus of *Macaca mulatta*, *Pongo abelii* and *Pan troglodytes. Human* is the consensus of 100 chromosomes, wherever it differs from the ancestral amino acid. *Weight* is derived from the evolutionary rate of exchange of each amino acid pair (for details see “Methods”). The table shows the amino acids in the constructs used in the experiments; current data show that there is indeed variation within modern humans at the signal peptide site. ^a^EC1, EC2, EC3: extracellular cadherin domains 1–3

Functional data show the importance of the distribution of the substitutions among the domains of the protein. Clustered protocadherins are proposed to serve as adhesion proteins that may regulate synaptic contacts between neurons [36, 37]. So far, the function of murine, but not human, clustered protocadherins has been tested in cell culture models and intact organisms [38–50]. In cell culture, among six extracellular cadherin repeats, one transmembrane and one cytoplasmic domain, the ones most important for protocadherin specificity are EC2 and EC3 [43], and nine of the changes in human PCDHB11 are concentrated in these two domains (Table 2), suggesting again that they might be relevant.

Very recently, crystal structures of the EC1-3 domains of several murine protocadherins, among them the β-protocadherin PCDHB1, have been published [51, 52]. By homology to the crystal structure of monomeric PCDHB1 EC1-3 [51], all ten human-specific amino acids in these domains of PCDHB11 are expected to be at least partly exposed to water; such surface-exposed amino acids are less constrained by the structure and may therefore be more variable, unless they contribute to dimer interfaces. In this regard, it is relevant to note that Thr185 in the PCDHB1 structure, corresponding to human-specific PCDHB11 Ser213, hydrogen bonds with Thr143, which was shown to be necessary for protocadherin dimerization in a cell-based assay [51]. Furthermore, the residue corresponding to human-specific PCDHB11 Ser134 contributes to crystal contacts in certain γ-protocadherins, and so do the EC2 β4-β5, the Phe-X_10_-Phe loop and the EC3 β7 loop, which in PCDHB11 are predicted to contain human-specific Ile185, Phe281 and His336, respectively [52].

While crystal contacts are not evidence of functional importance, and different clustered protocadherins may dimerize slightly differently, the homologies mentioned would suggest, on a purely structural basis, that some of the human-specific mutations might affect the adhesivity of PCDHB11. However, these putative conclusions from bioinformatical investigation depend on functional confirmation.

### Functional test of human PCDHB11

**Fig. 2:**
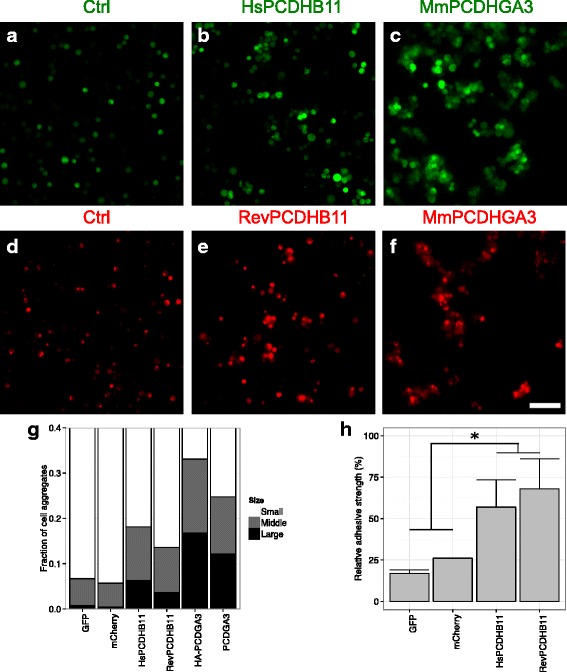
Homophilic adhesion of PCDHB11 and PCDHGA3 in human suspension cells. GFP was electroporated alone (a) or in combination with human PCDHB11 (b) or murine PCDHGA3 (c) into K562 cells; in the same way, mCherry was electroporated alone (d) or in combination with reverted PCDHB11 (e) or murine PCDHGA3 (f). Scale bar: 250 μm. g Fraction of large clusters (>30 cells), medium clusters (10–30 cells) or small clusters (<10 cells) in each condition. h Adhesive strength of cells in each condition as proportion of clusters with ≥ 10 cells, relative to the condition with the same fluorescent protein and PCDHGA3. Error bars: standard error of mean. The three levels of protocadherin (none, PCDHB11, PCDHGA3) were significantly different from each other (ANOVA, *p* = 0.0006; Tukey HSD test: none vs. PCDHB11, *p* = 0.036; none vs. PCDHGA3, *p* = 0.0005; PCDHB11 vs. PCDHGA3, *p* = 0.026; *n* = 4 transfections)

Following an established protocol [43], human PCDHB11 and a well-investigated murine control, PCDHGA3, were electroporated into the normally non-adhesive human suspension cell line K562. Murine PCDHGA3 caused the appearance of large cell clusters, as previously described (Fig. 2c, f); cells expressing human PCDHB11 formed clusters, but smaller ones (Fig. 2b). The proportion of larger cell clusters among all cells was quantified in four independent transfections; the adhesive strength conferred by human PCDHB11 was significant when compared to negative controls, but significantly lower than that conferred by murine PCDHGA3 (Fig. 2g, h).

**Fig. 3:**
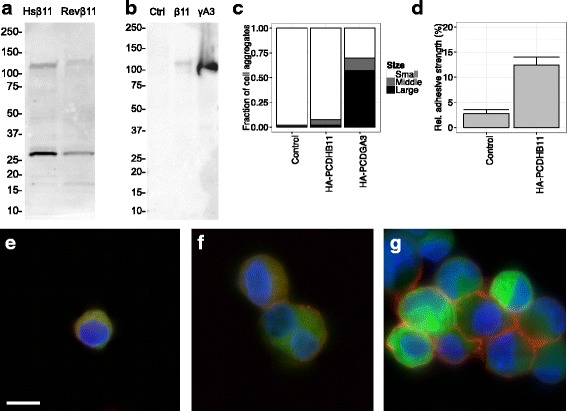
Expression of protocadherins. GFP was electroporated alone or in combination with protocadherins into K562 cells. a Immunoblotting of cell lysates with anti-GFP shows bands compatible with the expected molecular weights for GFP (27 kDa) and mature PCDHB11-GFP (110 kDa); image representative of three transfections. b Immunoblotting of cell lysates with anti-HA shows bands compatible with the expected molecular weights for mature HA-PCDHB11-GFP (113 kDa) and HA-PCDHGA3 (99 kDa); image representative of two transfections. c Proportion of medium and large clusters was lower in HA-PCDHB11-GFP-transfected than in HA-PCDHGA3-transfected cells, but higher than in control cells (*n* = 10-12 images per condition). d Proportion of medium to large cell clusters when normalized by HA-PCDHGA3. For visualization of surface protocadherins, live cells transfected with GFP alone (e) or in combination with HA-PCDHB11-GFP (f) or HA-PCDHGA3 (g) were stained with anti-HA-biotin and streptavidin-Alexa555, then fixed. *Blue:* DAPI. *Green:* GFP. *Red:* surface HA-tagged protocadherins. *Scale bar:* 10 μm

The weak adhesivity of PCDHB11-transfected cells might be explained by low expression levels. However, the expression of PCDHB11 fused to green fluorescent protein (GFP) was easily detected by Western Blot (Fig. 3a). It has been shown that N-terminal hemagglutinine (HA) tags, which allow selective staining of surface-exposed proteins, do not reduce the adhesion mediated by γ-protocadherins [43]. Reasoning that an N-terminal HA-tag would also preserve any adhesion mediated by PCDHB11, cells were transfected with HA-PCDHB11-GFP or murine HA-PCDHGA3. HA-PCDHB11-GFP was expressed at much lower levels (Fig. 3b), and this was reflected in lower surface expression (Fig. 3e-g) and a lower proportion of large cell clusters (Fig. 3c, d) than with HA-PCDHGA3. Nevertheless, PCDHB11 was expressed and reached the cell surface.

One reason for the low adhesivity of human PCDHB11 would be a loss of function as consequence of one or more of the mutations that occurred during human evolution. In order to test for an effect of the human-specific substitutions, a protein with reversion of these substitutions to the consensus sequence of non-human primate PCDHB11 proteins (Table 2) was synthesized and expressed in K562 cells. This reverted PCDHB11 had adhesive properties indistinguishable from modern human PCDHB11 (Fig. 2b, e, g, h). While it is not possible to exclude subtle changes, based on the present data, a complete loss or gain of adhesivity, due to the human-specific substitutions, can be refuted.

**Fig. 4:**
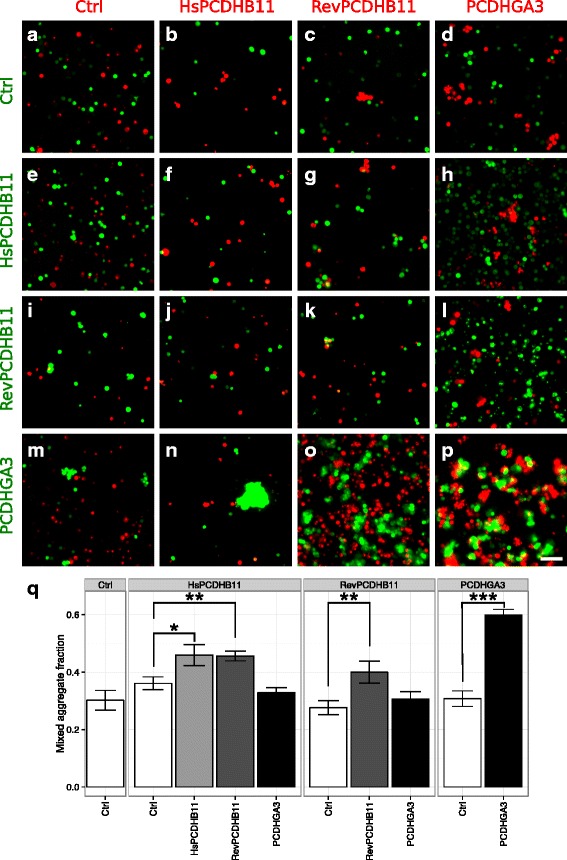
Specificity of adhesion of PCDHB11. Different populations of K562 cells were separately electroporated as in Fig. 2, where one population received GFP and a protocadherin, and the other mCherry and a protocadherin. Green and red populations were mixed 24-48 h later, rocked for 2 h and then photographed while still alive. a-p The identity of the protocadherins in the green and red populations are indicated. Scale bar: 250 μm. q For each pair of cell populations, the conditions with switched fluorophores were joined, and the proportion of mixed clusters (containing both *red and green cells*) was expressed as fraction of all clusters. In an overall 2-way ANOVA, the identity of the cell populations and their interaction were each highly significant (*n* = 17-84 images per condition, *p* < 10^- 8^). In each subpanel, the identity of the second cell population was significant (ANOVA, *p* < 0.05). In each subpanel, the second cell populations were significantly different from control where indicated (linear models with Bonferroni correction for comparison with control; *:*p* < 0,05; **: *p* < 0.01; ***: *p* < 0.001)

The properties of both reverted and modern human PCDHB11 were also investigated by quantifying co-clustering with other cells. Different populations of K562 cells were transfected with protocadherins and either green or red fluorescent protein, and the two populations were mixed on the following day. As expected, K562 cells without any protocadherin did not form clusters (Fig. 4a), nor did cells transfected with a protocadherin form mixed clusters with negative controls (Fig. 4b-d, e, i, m). Cells transfected with PCDHGA3 and PCDHB11, respectively, did not adhere to each other, which would manifest as a high proportion of mixed clusters (Fig. 4h, l, n, o). However, two cell populations, each transfected with murine PCDHGA3, adhered strongly to each other, as expected (Fig. 4p). In mixtures of modern and ancestral PCDHB11-expressing cells, some mixed clusters were observed (Fig. 4f, g, j, k), which were smaller than the PCDHGA3 clusters. The co-occurrence of green and red cells in the same clusters was quantified in each condition (Fig. 4q). PCDHB11-expressing cells adhered more to each other than to negative controls or to murine PCDHGA3. The human-specific changes did not abolish the weak mutual adhesion.

### Intra-human variability in PCDHB11

**Fig. 5:**
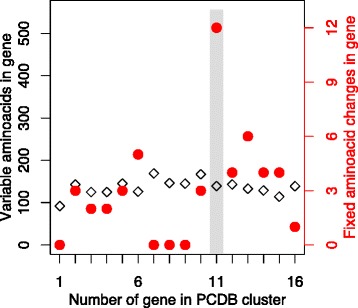
Intra-human variability of PCDHB11 is not elevated. For each protein in the human *PCDHB* cluster, the number of positions subject to intra-human variability is shown as *black open diamonds* (scale on left axis) and the number of substitutions on the human lineage as *red full circles* (scale on right axis, same data as in Table 1). *Shaded grey bar:* PCDHB11

Since these assays did not reveal a change in the adhesion function of PCDHB11, the high number of human-specific amino acid substitutions in *PCDHB11* might be thought to be due to a higher local mutational burden or reduced purifying selection. Such processes would necessarily increase variability among humans. However, it has been reported before, in an ethnically homogeneous sample, that human PCDHB11 had lower variability than other β-protocadherin genes [53]. Here, the numbers of non-synonymous minor alleles in all human β-protocadherins were reanalyzed using dbSNP; they ranged from 92 to 167 variable positions, and the number for PCDHB11 was 139, coincidentally the median of the distribution (Fig. 5, open diamonds). This normal intra-human variability of PCDHB11 stands in contrast to the evolutionary data, where PCDHB11 has a much higher number of human-specific amino acid substitutions than any other β-protocadherin (Table 1; Fig. 5, closed circles).

## Discussion

The aim of this work was to search systematically for the brain protein that has changed the most during human evolution and that might contribute to uniquely human brain function and pathology [23]. Evolutionary studies, such as the present one, might help to pinpoint molecules important for human functioning [54]. The most promising candidate from the bioinformatical part of the work was the β-protocadherin PCDHB11.

The β-protocadherin cluster, as a whole, was shown to be rapidly diverging between humans and chimpanzees [24]. According to the Allen Brain Atlas [55], PCDHB11 mRNA is expressed in the human brain, especially in the hippocampus, striatum, substantia nigra and locus ceruleus [56]. Human PCDHB11 has no direct murine homolog [57], but murine β-protocadherin mRNAs are expressed combinatorially in Purkinje cells [58]. At the protein level, some murine β-protocadherins have been shown to be expressed in synapses of the central nervous system [59, 60]. With regard to the physiological importance of β-protocadherins, the only data in humans so far have revealed a very high expression of PCDHB11 and PCDHB13 on the melanoma cell surface [61, 62]. Hypermethylation of promoters in the protocadherin clusters, collectively, which is expected to downregulate gene expression, is a signal of Wilms’ tumor [63], breast cancer [64] and neuroblastoma [65–67].

The most widely accepted model for the operation of clustered protocadherins is homophilic adhesion, where a hetero-oligomer of α-, β- and/or γ-protocadherins on the surface of one cell binds to an oligomer of the exact same composition on the surface of another cell [43, 48, 51]. While this homophilic binding can be measured as the formation of cell clusters in the K562 cell line model, in neurons it is proposed to lead to synapse disruption and therefore dendritic self-avoidance, optimizing the coverage of a neuron’s territory [46, 51]. The present data extend this established cell culture model for the first time to a human protocadherin. Human PCDHB11 does induce the formation of K562 cell clusters, but at a very low level, and this low adhesivity has apparently been characteristic of PCDHB11 since before the divergence of human and chimpanzees. It is unclear if such a low level of adhesivity has functional relevance in the intact organism.

As homophilic adhesion is the main function of clustered protocadherins investigated experimentally so far, these results discourage a functional interpretation of the human-specific substitutions and require considering neutral evolution of this locus [68]. However, the unremarkable intra-human variability of *PCDHB11* suggests that there is no mutational hot spot at work, nor have selection constraints on *PCDHB11* been specially relaxed. No exon of *PCDHB11* was found to have high levels of biased gene conversion [69]. Moreover, while gene conversion events in certain human protocadherins have been reported, these events concentrated on the EC6 and cytoplasmic domains, sparing the EC1-EC3 domains [70]. Nine of twelve changes in PCDHB11 are located in EC2 and EC3, and it has been suggested, in a joint analysis of all human protocadherins, that positively selected positions are concentrated in these domains [71]. Finally, we note that the lack of increased intra-human variability also seems to discourage the hypothesis of diversifying selection.

While some other neutral process may yet explain the high density of human-specific substitutions in *PCDHB11*, it is also possible that selection might have occurred on an as yet unknown function of human PCDHB11. The subcellular localization of chick α-protocadherins [72] and rodent and primate β-protocadherins [59, 60] was suggested to be incompatible with the commonly assumed homophilic adhesion function. While no data on possible trans-interaction partners of β-protocadherins are available, heterophilic adhesion to integrin has been proposed for murine α-protocadherins [49], and additional intracellular roles distinct from cell adhesion have been suggested for some murine γ-protocadherins [39, 41, 42].

## Conclusion

The strategy employed here resulted in the discovery of *PCDHB11* as a candidate gene for positive selection on the human lineage, combining a high number of potentially relevant substitutions on the human lineage with low intra-human variability. In spite of these genetic results, cell-based tests on the only proposed function for PCDHB11 did not indicate a functional change. If the molecular bases of human cognitive capacities can indeed be pinpointed to specific parts of the genome, they may well be discovered in changes affecting expression levels, such as copy number variations or non-coding regulatory sequences.

## Methods

### Bioinformatics

A list of genome positions where a panel of 100 human haploid genomes agrees on one base, while the reference genomes of common chimpanzee (*Pan troglodytes*), orangutan (*Pongo abelii*) and rhesus macaque (*Macaca mulatta*) agree on a different base, was kindly supplied by Martin Kircher [34]. For the existing sequence data, such a procedure was considered more prudent than classification of amino acid positions into different variability classes [73], both because of the small phylogenies and because structural information is not available for all proteins in the set. Human proteins where this base difference resulted in an amino acid difference were retained for further analysis if they were associated with the Gene Ontology terms [74]:

1. “integral component of plasma membrane” or “anchored component of external side of plasma membrane”, and additionally
2. any term beginning with “nervous”, “neuron”, “dendr”, “axon” or “synap” (except for those containing “enteric”, “autonomous”, “synaptonem”, “axonem” or “dendritic cell”).

Since the surface annotation in this data base was incomplete, all members of a protein family (defined as those proteins having UNIPROT codes [75] beginning with the same three letters) were included if at least one member had passed the cell surface filter. The 329 resulting proteins were ranked by number of amino acid substitutions and also by number of substitutions normalized to the length of the protein.

The BLOSUM100 matrix gives the rate of one amino acid being exchanged for another one when calibrated for protein sequences of >99 % identity [76], corresponding to the overall identity between human and chimpanzee proteins [24]; the matrix was converted into weights, so that the most common exchanges had a weight of 3 and the least common exchange a weight of 15. These weights were added up for all differences in each protein. Furthermore, the weighted number of differences was normalized by the length of the protein. To test the reliability of the ranking, the same procedure was repeated with the BLOSUM62 matrix (which is more commonly used) and with the JTT matrix (which was suggested by PROTTEST [73] as the best model for an evolutionary tree of PCDHB11), and changing the matrix did not strongly affect the ranking. All analyses were done using custom-written Python scripts.

For examination of surface exposure, human PCDHB11 and all protocadherins discussed in references [51, 52] were aligned using Clustal Omega [77]. The amino acids that correspond to human-specific PCDHB11 substitutions were localized in structure 4ZPL (murine PCDHB1, the nearest homolog that has been crystallized), 4ZI9 (murine PCDHGC3) and 4ZI8 (murine (PCDHGA1).

### Molecular biology

Human PCDHB11 cDNA was ordered from imaGenes (Berlin, Germany), amplified by PCR using primers containing N*he*I and B*am*HI sites, and cloned into pEGFP-N1, so that GFP was added in frame at the C-terminus. HA-tagged PCDHB11-GFP was constructed by cloning annealed oligonucleotides coding for the HA-tag YPYDVPDYAE (Lifetech, São Paulo, Brasil) after the signal peptide cleavage site of PCDHB11. A reverted version was constructed by substituting the 12 consensus bases of the other primates into the human PCDHB11-GFP sequence, which was then ordered from Genscript (Piscataway, NJ). The plasmids pcDNA3-HA-*Mm*PCDGA3 and pmRFP-*Mm*PCDGA3, coding for *Mus musculus γ*-protocadherin A3, were a kind gift from Dr. Dietmar Schreiner. All constructs were checked by Sanger sequencing of the entire open reading frame (Sequencing Core Facility, Institute of Biophysics, Federal University of Rio de Janeiro).

The number of variable positions in modern humans was calculated for each member of the *PCDHB* cluster as the sum of missense, nonsense, stop lost and frame shift mutations in dbSNP [78].

### Cell culture

K562 cells, an immortalized cell line derived from human leukemia [79], were a kind gift of Martin Bonamino, National Institute for Cancer Research, Rio de Janeiro, Brasil. K562 cells were cultured in a rich medium [80], and 2 · 10^6^ cells were electroporated with 1 μg of pEGFP-N1 or membrane-anchored mCherry plus 5 μg of the indicated protocadherin plasmid DNA, using “1 M” buffer and Mirus Biotech (Madison, WI) or Bio-Rad (São Paulo, Brasil) cuvettes on a Nucleofector II (Lonza, Basel, Switzerland) [80]. Immediately after electroporation, cells were resuspended in 1 ml of the culture medium and diluted to 8 ml in warm culture medium. On the following day, where indicated, cells from different electroporation conditions were mixed 1:1 in 6-well plates. The plates were rocked for at least 2 h at 37 °C and 5–6 movements per minute, and 5–10 fields of view in each well were photographed on an Eclipse TE300 microscope using a 10x magnifying objective and a DS-QiMC camera (Nikon, Melville, NY).

For immunoblotting, transfected cells were centrifuged 10 min at 200 g, 37 °C, and the supernatants were resuspended in radioimmunoprecipitation assay (RIPA) buffer including protease inhibitors (Thermo Fisher Scientific, Waltham, MA). 100 μg (for GFP) or 20 μg (for HA) of total protein were applied per well of a 6-15 % gradient Tris-Glycine gel (Bio-Rad Laboratories, Hercules, CA), and after eletrophoresis transferred to a nitrocellulose membrane (GE Healthcare, Little Chalfont, UK). For GFP detection, membranes were incubated with a rabbit polyclonal antibody (Life Technologies, A-11122) diluted 1:2000 in 50 mM Tris, 100 mM NaCl, 0.1 % Tween 20, pH 7.4, with 3 % bovine serum albumine (Sigma, St. Louis, MO) and a horse-radish peroxidase-conjugated anti-rabbit secondary antibody (Invitrogen, Waltham, MA), while for HA revelation, they were incubated with biotinylated 3 F10 rat monoclonal antibody (Roche Life Science, Indianapolis, IN) diluted 1:500 in the same buffer, and streptavidin-horseradish peroxidase (Invitrogen, Waltham, MA). All membranes were revealed on a ChemiDoc system (Bio-Rad) using Super Signal West Femto (Thermo Fisher Scientific).

For HA staining, live K562 cultures were incubated for one hour with the biotinylated 3 F10 anti-HA, then for 40 min with streptavidin-Alexa555, both diluted 1:250 in culture medium at 8 °C, then fixed for 15 min in 4 % paraformaldehyde, 4 % sucrose in phosphate-buffered saline at 8 °C, deposited on slides using a CytoSpin (Thermo Fisher Scientific) and mounted in ProLong containing 4′,6-diamidino-2-phenylindole (DAPI; Invitrogen). Images were taken on an Axiovert 200 M microscope, using a 100x objective (Zeiss, Göttingen, Germany).

### Quantification

For quantification of aggregate size, cell clusters were thresholded in ImageJ 2.0.0 [81] using the triangle algorithm implemented in the AutoThreshold plugin for ImageJ [82], and all areas of at least 100 pixels were exported for analysis in R [83]. From the size of a single cell, defined as the mode cluster size of 150 pixels for control GFP-transfected cells, the cutoff for clusters of 10 cells was calculated as 696 pixels (assuming spherical clusters), and for clusters of 30 cells as 1448 pixels. For statistical analysis, the fraction of clusters containing at least 10 cells was calculated for each condition and day. After normalizing each condition on each day to the PCDHGA3 condition on the same day with the same fluorophore, there was no significant difference in fraction of medium and large clusters between human and reverted PCDHB11 (*n* = 4 transfections). The two control conditions, the two PCDHB11 conditions and the two PCDHGA3 conditions were therefore pooled for further analysis.

For quantification of adhesion specificity, the separate green and red channels of each field were auto-thresholded as above, and the two masks were added. The presence of thresholded green and red cells was recorded for each cluster. In R, the proportion of clusters containing both green and red cells, relative to all clusters, was calculated for each image.

## Ethics

In vitro research using already derived and established human cell lines such as the immortalized K562 cells used in this publication, from which the identity of the donor(s) cannot readily be ascertained by the investigator, are not considered human subject research, and institutional review is not required for such research.

## Consent to publish

Not applicable.

## Availability of data and materials

No human or animal samples were sequenced in this work. The dataset of human sequences supporting the conclusions of this article is freely available in the European Nucleotide Archive, accession number ERP000125 [84].

## Abbreviations

DAPI: 4′,6-diamidino-2-phenylindole
EC1-6: extracellular cadherin domain 1–6
GFP: green fluorescent protein
HA: hemagglutinin tag
